# The variation of the burden of hypertension and diabetes in two large districts of the city of São Paulo, Brazil, based on primary health care routinely-collected data

**DOI:** 10.1371/journal.pone.0213998

**Published:** 2019-03-15

**Authors:** João Luiz Miraglia, Ana Carolina Cintra Nunes Mafra, Camila Nascimento Monteiro, Luciana Morais Borges

**Affiliations:** 1 Programas Governamentais, Instituto Israelita de Responsabilidade Social, Hospital Israelita Albert Einstein, São Paulo, São Paulo, Brasil; 2 Departamento de Medicina Preventiva, Faculdade de Medicina, Universidade de São Paulo, São Paulo, São Paulo, Brasil; Temple University, Lewis Katz School of Medicine, UNITED STATES

## Abstract

**Background:**

Noncommunicable diseases (NCDs) were responsible for 72.3% of global deaths in 2016, with cardiovascular diseases accounting for almost half of those deaths and low- and middle-income countries carrying the biggest burden. As a result, the prevention and control of NCDs is recognized as urgent, while better surveillance at the country level could result in more effective policies. Hence, the objective of this study was to obtain more detailed information on the distribution of the prevalence of hypertension and diabetes among the population of two large districts of the city of São Paulo in Brazil, and to compare these findings to the results of a citywide health survey.

**Methods and findings:**

This cross-sectional study used primary health care (PHC) routinely-collected data. The study population included 187,110 individuals 20 years of age or older registered in 13 public PHC facilities at two districts of the city of São Paulo in 2015. Data extracted from SIAB, a primary care database, was used to calculate age and sex directly standardized prevalences for diabetes and hypertension for each PHC facility. The prevalence of hypertension among women was significantly higher than the prevalence among men in the entire study population, and in every PHC facility. There was great variation among PHC facilities that was more pronounced among women. The prevalence of diabetes among women was significantly higher than the prevalence among men in the entire study population, and in every PHC facility, but there was little variation among PHC facilities.

**Conclusions:**

This study provided information that could help with policy planning and allocation of resources, and demonstrated the use of PHC routinely-collected data to generate important insights that if replicated could have a substantial impact given the broad coverage of the national public PHC program in Brazil.

## Introduction

Noncommunicable diseases (NCDs) were responsible for 72.3% of global deaths in 2016, with cardiovascular diseases (CVDs) accounting for almost half of those deaths. Between 2006 and 2016, these numbers increased by 16.1% for NCDs and by 14.5% for CVDs, while low- and middle-income countries (LMICs) carried the biggest burden with the majority of deaths and premature deaths [1,2].

Cardiovascular diseases and their associated risks factors are also leaders in terms of disability-adjusted life years (DALYs) worldwide. In 2016, high systolic blood pressure (SBP) was the leading risk factor in terms of attributable DALYs, with ischemic heart disease (IHD) as its largest source, followed by hemorrhagic stroke and ischemic stroke. High fast plasma glucose (FPG) was the third-leading risk factor in terms of attributable DALYs, with diabetes as its largest source, followed by IHD and chronic kidney disease [3].

In Brazil, the shift of disease burden from infectious to NCDs followed important structural and economic changes over the past 50 years, with rapid demographic and nutritional transitions driven by greater income, industrialization, improved access to food, urbanization, and globalization of unhealthy habits exposing the population to different risks associated with chronic diseases [4]. As a result, between 1990 and 2016, IHD moved from fourth to first among the leading causes of years of life lost (YLLs) surpassing diarrheal diseases. In addition, in 2016 high SBP and FPG were the second and fourth greatest contributors to DALYs among women, respectively, and high SBP was the second greatest contributor among men. These risk factors contributed primarily to DALYs from CVDs and diabetes, among others [5].

The prevention and control of NCDs is recognized as urgent, and as a consequence the World Health Organization established the Global Action Plan for the Prevention and Control of NCDs 2013–2020, which included among its six policy options and objectives surveillance and monitoring [6]. Better NCDs surveillance at the country level could provide more comprehensive health information resulting in improved and more effective policies and programs targeted at disease prevention.

Hence, the objective of this study was to obtain more detailed information on the distribution of the prevalence of hypertension and diabetes among the population of two large districts of the city of São Paulo in Brazil, and to compare these findings to the results of a citywide telephone-based health survey.

## Methods

### Population and setting

The source population included people living at the Campo Limpo and Vila Andrade districts at the city of São Paulo, Brazil, in 2015, representing a total of 368,133 individuals [7]. Although the state of São Paulo has the highest development index in the country [5], there is great variability among and within cities in the state. The city of São Paulo in particular has considerable, persistent and rising income disparities, and both districts included in the study have areas of high levels of vulnerability [8].

The Brazilian health system is made up of a public-private mix with three interconnected subsectors: the public national health system (Sistema Único de Saúde or SUS), the private (for-profit and nonprofit), and the private health insurance subsectors, and individuals can use services in all three [9]. All publicly financed health services and most common medications are universally accessible and free of charge for all citizens, and since 1994 the Family Health Program (now called the Family Health Strategy, or FHS) has reorganized primary health care (PHC) on the public sector [10]. As a result, the area of the two districts is covered by 14 public PHC facilities, from which 13 were included in the study. These facilities comprise 87 FHS teams composed by a physician, a nurse, tow nurse assistants, and 6 community health workers that are organized geographically covering populations of up to 1,000 households each, with no overlap or gap between catchment areas [10].

The database population included every individual registered in the 13 PHC facilities included in the study, while the study population was limited to individuals 20 years of age or older.

### Methods

The study was approved by the São Paulo Municipal Health Department ethics committee (CAAE 84531418.6.3001.0086), and by the Hospital Israelita Albert Einstein ethics committee (CAAE 84531418.6.0000.0071).

This was a cross-sectional study that used PHC routinely-collected data.

The distribution of hypertension, diabetes, age and sex for the study population were obtained for each PHC facility with data extracted from SIAB [11], a PHC database managed by the local FHS teams and used to help in strategic planning and management of local health systems. SIAB includes data on families, housing and sanitation conditions, health status, productivity and composition of health teams. Individuals included in the database had their diagnosis of hypertension and diabetes confirmed by a physician.

Age and sex directly standardized prevalences were calculated for diabetes and hypertension for each PHC facility with the population of the entire city of São Paulo as the reference [7]. Four age brackets (20–39, 40–49, 50–59 and ≥60 years of age) were provided by the SIAB database, and were used for the calculations. Ninety-five percent confidence intervals (CIs) were calculated to the directly standardized prevalences following a method described previously [12].

The prevalences of hypertension and diabetes, and their respective 95% CIs, for the entire city of São Paulo were obtained from the Telephone Surveillance of Risk and Protective Factors for Chronic Diseases (VIGITEL), conducted by the Brazilian Ministry of Health in 2015 [13]. This was a national telephone-based health survey with a probabilistic samples of the adult population (18 years of age and older) residing in households served by at least one landline telephone. It was conducted in all capitals of the 26 Brazilian states and the Federal District, and included self-reported diagnosis of hypertension and diabetes.

The analyses were conducted with the R software environment for statistical computing and graphics, version 3.4.1 [14], and the study followed the REporting of studies Conducted using Observational Routinely-collected health Data (RECORD) Statement [15].

## Results

The age distribution by sex for the city of São Paulo, the entire study population, and each of the 13 PHC facilities can be found in Table 1. The study included 187,110 individuals, representing 72.6% of the total population 20 years of age or older living in the area, with a median number of individuals registered at the PHC facilities of 13,024 (p25 = 12,046; p75 = 16,169). The sex distribution was similar among the city of São Paulo, the entire study population and the PHC facilities, with a little more than 50% of women in each one of those groups. The age distribution for the 40–59 years of age bracket was similar between the entire study population and the city of São Paulo. Higher percentages in the 20–39 year of age bracket, and lower percentages in the ≥60 years of age bracket, for women and men, were found in the entire study population when compared to the city of São Paulo. There was more variation among PHC facilities in the age distribution, for women and men, in the 20–39 and ≥60 years of age brackets. The percentage among women for the 20–39 and ≥60 years of age brackets ranged from 24.3% to 34.0% (median = 26.9%; p25 = 25.6%; p75 = 28.6%), and from 3.2% to 11.8% (median = 8.6%; p25 = 7.4%; p75 = 10.6%), respectively. The percentage among men for the 20–39 and ≥60 years of age brackets ranged from 22.0% to 30.3% (median = 25.0%; p25 = 22.6%; p75 = 26.0%), and from 2.2% to 7.5% (median = 5.6%; p25 = 4.8%; p75 = 6.7%), respectively.

**Table 1:** Age distribution by sex for the city of São Paulo, total study population and PHC facilities of individuals 20 years of age and older.

	Women (%)				Men (%)				
	Years of age								
	20–39	40–59	≥60	Total	20–39	40–59	≥60	Total	N
City of São Paulo	23.7	19.0	11.0	53.7	22.4	16.4	7.5	46.3	8455125
Total study population	27.7	18.5	8.3	54.5	24.9	15.0	5.6	45.5	187110
PHC facility									
A	26.9	18.3	8.6	53.8	24.4	15.6	6.2	46.2	21948
B	24.9	19.1	10.9	54.9	22.5	15.4	7.1	45.1	19829
C	28.3	18.2	8.1	54.6	25.3	14.6	5.6	45.4	19237
D	25.2	19.2	10.9	55.3	22.6	15.5	6.7	44.7	16169
E	26.8	18.4	8.6	53.7	24.3	15.6	6.4	46.3	16052
F	25.6	19.6	10.6	55.8	22.0	15.2	6.9	44.2	13399
G	24.3	19.1	11.8	55.2	22.1	15.0	7.6	44.8	13024
H	28.0	18.6	7.7	54.4	26.0	14.8	4.8	45.6	12803
I	26.9	18.8	8.7	54.5	25.1	14.9	5.5	45.5	12135
J	32.6	17.2	3.2	53.0	30.3	14.4	2.3	47.0	12046
K	33.0	17.2	3.9	54.2	29.2	13.8	2.8	45.8	11626
L	34.0	17.1	3.2	54.4	29.1	14.3	2.2	45.6	10291
M	28.6	19.0	7.4	55.0	25.0	15.2	4.8	45.0	8551

PHC, primary health care; N, total number.

**Fig 1:**
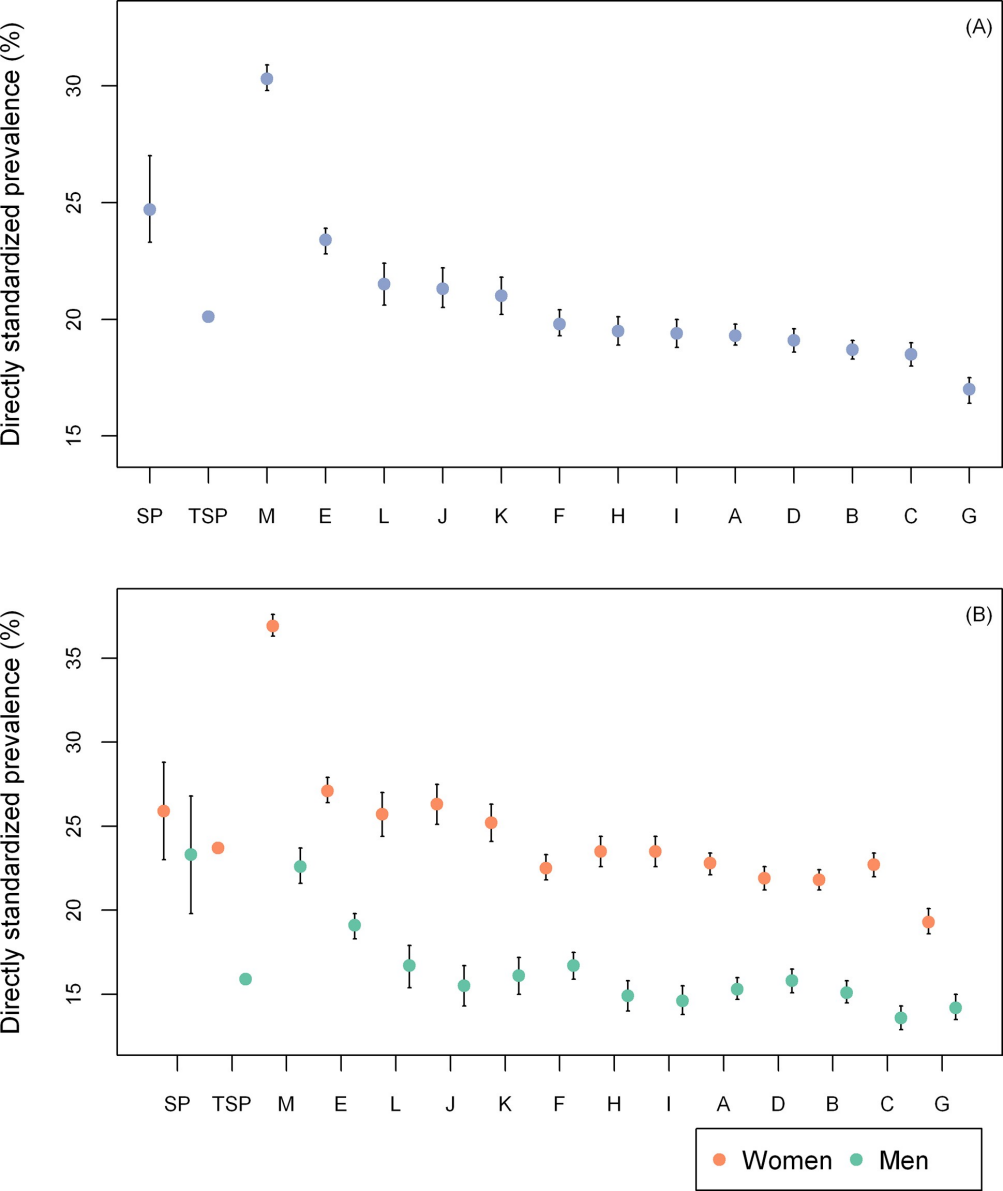
Age and sex directly standardized prevalences of hypertension. (A) Not stratified by sex. (B) Stratified by sex. SP, City of São Paulo/VIGITEL Brasil 2015; TSP, total study population.

The prevalence of hypertension in the entire study population was significantly lower than the prevalence in the city of São Paulo (Table 2 and Fig 1), however there was great variation among PHC facilities. Prevalences among PHC facilities ranged from 17.0% to 30.3% (median = 19.5%; p25 = 19.1%; p75 = 21.3%), with significant differences. When stratified by sex, the prevalence of hypertension in the entire study population was significantly lower than the prevalence in the city of São Paulo for men but not for women. Furthermore, the prevalence of hypertension among women was significantly higher than the prevalence among men in the entire study population, and in every PHC facility, but not for the city of São Paulo. The difference between women and men in the entire study population was 7.8%, and ranged from 5.1% to 14.3% (median = 8.6%; p25 = 6.7%; p75 = 9.1%) among PHC facilities. The highest prevalence of hypertension for the PHC facilities was found among women with 36.9%, while men had 22.6% as their highest prevalence. The top five highest prevalences of hypertension for the PHC facilities among women were significantly higher than the highest prevalence among men. Women also presented greater variation among PHC facilities, with prevalences of hypertension ranging from 19.3% to 36.9% (median = 23.5%; p25 = 22.5%; p75 = 25.7%), while the prevalence among men ranged from 14.2% to 22.6% (median = 15.5%; p25 = 14.9%; p75 = 16.7%).

**Table 2:** Age and sex directly standardized prevalence of hypertension with the city of São Paulo as the standard population.

	% (IC 95%)		
	Total	Women	Men
City of São Paulo^a^	24.7 (23.3–27.0)	25.9 (23.0–28.8)	23.3 (19.8–26.8)
Total Study population	20.1 (19.9–20.3)	23.7 (23.5–24.0)	15.9 (15.7–16.1)
Public PHC unit			
M	30.3 (29.8–30.9)	36.9 (36.3–37.6)	22.6 (21.6–23.7)
E	23.4 (22.8–23.9)	27.1 (26.4–27.9)	19.1 (18.3–19.8)
L	21.5 (20.6–22.4)	25.7 (24.4–27.0)	16.7 (15.4–17.9)
J	21.3 (20.5–22.2)	26.3 (25.1–27.5)	15.5 (14.3–16.7)
K	21.0 (20.2–21.8)	25.2 (24.1–26.3)	16.1 (15.0–17.2)
F	19.8 (19.3–20.4)	22.5 (21.8–23.3)	16.7 (15.9–17.5)
H	19.5 (18.9–20.1)	23.5 (22.6–24.4)	14.9 (14.0–15.8)
I	19.4 (18.8–20.0)	23.5 (22.6–24.4)	14.6 (13.8–15.5)
A	19.3 (18.9–19.8)	22.8 (22.1–23.4)	15.3 (14.7–16.0)
D	19.1 (18.6–19.6)	21.9 (21.2–22.6)	15.8 (15.1–16.5)
B	18.7 (18.3–19.1)	21.8 (21.2–22.4)	15.1 (14.5–15.8)
C	18.5 (18.0–19.0)	22.7 (22.0–23.4)	13.6 (12.9–14.3)
G	17.0 (16.4–17.5)	19.3 (18.6–20.1)	14.2 (13.5–15.0)

CI, confidence interval; PHC, primary health care.

^a^VIGITEL Brasil 2015.

**Fig 2:**
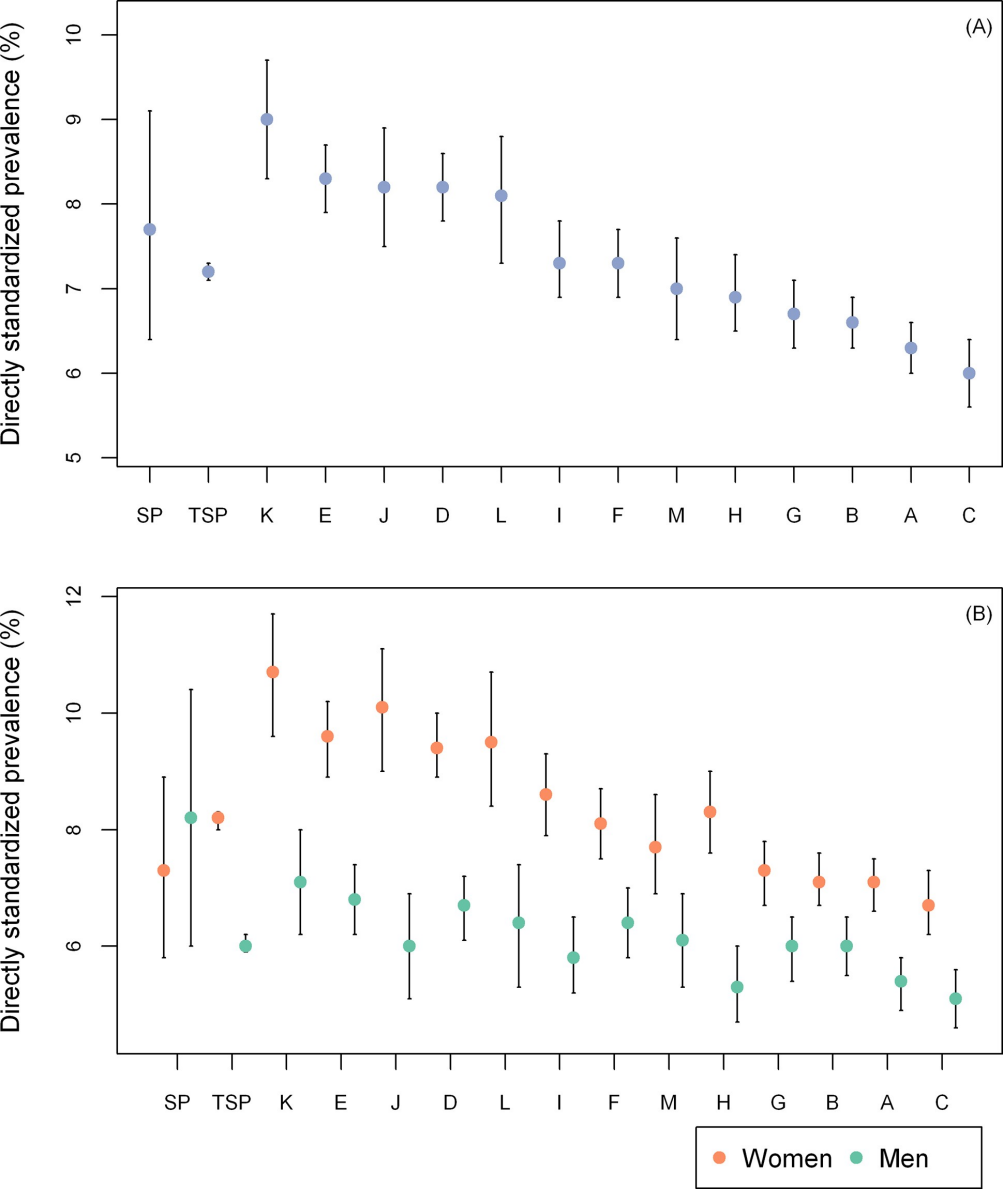
Age and sex directly standardized prevalence of diabetes. (A) Not stratified by sex. (B) Stratified by sex. SP, City of São Paulo/VIGITEL Brasil 2015; TSP, total study population.

The prevalence of diabetes among the entire study population was similar to the prevalence in the city of São Paulo (Table 3 and Fig 2), and unlike what was found for hypertension, there was little variation among PHC facilities, with prevalences ranging from 6.9% to 9.0% (median = 7.3%; p25 = 6.7%; p75 = 8.2%). There were no significant differences between the entire study population and the city of São Paulo when prevalences of diabetes were stratified by sex. Similar to what was found for hypertension, the prevalence of diabetes among women was significantly higher than the prevalence among men in the entire study population and in every PHC facility, but not in São Paulo. However, the differences found were smaller with 2.2% in the entire study population, and ranging from 1.1% to 4.1% (median = 2.7%; p25 = 1.6%; p75 = 3.0%) among PHC facilities. The highest prevalence of diabetes among PHC facilities was also found among women with 10.7%, while men had 7.1% as their highest prevalence. The top five highest prevalences of diabetes in the PHC facilities among women were also significantly higher than the highest prevalence of diabetes among men, with greater variation also found among women. However, the variation was smaller than the one found for hypertension, with prevalences ranging from 6.7% to 10.7% (median = 8.3%; p25 = 7.3%; p75 = 9.5%) for women and from 5.1% to 7.1% (median = 6.0%; p25 = 5.8%; p75 = 6.4%) among men.

**Table 3:** Age and sex directly standardized prevalence of diabetes with the city of São Paulo as the standard population.

	% (CI 95%)		
	Total	Women	Men
City of São Paulo^a^	7.7 (6.4–9.1)	7.3 (5.8–8.9)	8.2 (6.0–10.4)
Total Study population	7.2 (7.1–7.3)	8.2 (8.0–8.3)	6.0 (5.9–6.2)
Public PHC unit			
K	9.0 (8.3–9.7)	10.7 (9.6–11.7)	7.1 (6.2–8.0)
E	8.3 (7.9–8.7)	9.6 (8.9–10.2)	6.8 (6.2–7.4)
D	8.2 (7.8–8.6)	9.4 (8.9–10.0)	6.7 (6.1–7.2)
J	8.2 (7.5–8.9)	10.1 (9.0–11.1)	6.0 (5.1–6.9)
L	8.1 (7.3–8.8)	9.5 (8.4–10.7)	6.4 (5.3–7.4)
F	7.3 (6.9–7.7)	8.1 (7.5–8.7)	6.4 (5.8–7.0)
I	7.3 (6.9–7.8)	8.6 (7.9–9.3)	5.8 (5.2–6.5)
M	7.0 (6.4–7.6)	7.7 (6.9–8.6)	6.1 (5.3–6.9)
H	6.9 (6.5–7.4)	8.3 (7.6–9.0)	5.3 (4.7–6.0)
G	6.7 (6.3–7.1)	7.3 (6.7–7.8)	6.0 (5.4–6.5)
B	6.6 (6.3–6.9)	7.1 (6.7–7.6)	6.0 (5.5–6.5)
A	6.3 (6.0–6.6)	7.1 (6.6–7.5)	5.4 (4.9–5.8)
C	6.0 (5.6–6.4)	6.7 (6.2–7.3)	5.1 (4.6–5.6)

CI, confidence interval; PHC, primary health care.

^a^VIGITEL Brasil 2015.

## Discussion

The epidemiological transition in Brazil resulted in increased life expectancy and decreased mortality, however the overall burden of chronic conditions, including hypertension and diabetes, is growing [16]. The impact in São Paulo is clear, as it had very low rates of YLLs in 2016, when compared with other states, but one of the highest rates of years lost due to disability (YLDs) [16].

Given the impact of NCDs, the Sustainable Development Goals (SDGs) included the specific target of reducing premature mortality from NCDs by a third by 2030 [17], what could be achieved through the implementation of a set of well stablished cost-effective interventions [18]. Investments in CVD prevention and control has been shown to provide a very high economic return [19], and in Brazil, the FHS has been associated with reduced mortality from stroke and heart disease [20], evidence that supports the strengthening of health systems to prevent and control NCDs.

Quantifying risks to health and thus the targets of many public health actions is an essential prerequisite for effective public health [3], yet the data needed to assess the exact needs remains limited [19], making studies that evaluate in more detail geographic-specific burden of disease invaluable. In addition, the use of routinely-collected health data to that objective has the advantages of maximizing representativeness and generalizability, since data is collected under real-world circumstances, and of minimizing costs and effort [21].

Another aspect that makes more detailed local data valuable is the identification of variation, which could raise hypotheses and guide evaluations to better understand associated causes. The present study identified a meaningful difference in the prevalence of hypertension and diabetes between women and men, that had not been identified by the citywide telephone-based survey. Part of these differences could be explained by the known underutilization of health services, including blood glucose and blood pressure screening, by men [22]. On the other hand, CVDs cause one third of all deaths among women worldwide, and when compared to men with the same CVD risk, women are less likely to receive guidance on preventative treatments, to be prescribed statin therapy, and when prescribed, to achieve optimal targets and to adhere to these therapies [23]. So, further investigation is needed to clarify these differences in order to better inform policies to promote CVD prevention among men and women.

Variation in NCDs burden had already been identified within Brazilian states and cities [24] and was also found among the PHC facilities included in the study, being more evident among women and for hypertension. A recent review found strong evidence in LMICs supporting a positive association between low-income, low socioeconomic status, or low educational status and NCDs, including hypertension and diabetes [25]. This association could help to explain the variation among PHC facilities, since both districts included in the study have areas of high levels of vulnerability. Further evaluation of these findings would be important, since the reduction of health inequalities has been shown to improve socioeconomic outcomes for households, which in turn enhances health at the population and household levels [26].

Only 61% of households in Brazil have a landline telephone, with a heterogeneous distribution among cities [27], and as a result VIGITEL is known to overestimate the prevalence of hypertension [27,28]. This is in accordance with the lower prevalence of hypertension found among the entire study population, when compared to the survey estimates to São Paulo, what could have been further influenced by the fact that the study used diagnoses of hypertension established by a physician, while VIGITEL used self-reported diagnoses. However, when stratified by sex the prevalence of hypertension in the entire study population when compared to the city of São Paulo was lower among men but was similar among women, what could reinforce the explanation of underutilization of health services by men. Furthermore, no such differences were found in the prevalence of diabetes when the entire study population and the city of São Paulo were compared, and since the active surveillance of hypertension and diabetes performed by the FHS teams are closely related underutilization by men, in addition to losses to follow-up or database quality issues, appear less likely to be the cause of the more noticeable variation in the prevalence of hypertension. Thus, the lower overall study prevalence and the variation identified could be a better representation of the study population, demonstrating the value of routinely-collected health data.

This study is subject to the limitations of observational studies and of routinely-collected health data, with the possibility of the interference by multiple errors and biases (e.g., data linkage problems, misclassification bias and underreporting) [21].

### Conclusions

Health is an important driver of the SDGs and the reduction of NCDs is key in the promotion of this agenda, since it could have an impact in many other goals like poverty, inequality and economic growth, as they intersect with each other through NCDs [29].

This study provided information about the size and distribution of the prevalences of hypertension and diabetes in two large districts of São Paulo, and could help with policy planning and deriving greater value for allocated resources. Moreover, the study results provide an opportunity to inform potential strategies for FHS teams and other health care workers to advocate on behalf of their population. Finally, this study also demonstrated the use of PHC routinely-collected data to generate important insights, what could promote its replication in many parts of the world that also have implemented similar PHC programs, while in Brazil it could have a substantial impact, given the size and coverage of the FHS with 40,510 teams serving 125 million people (64% of the population) in 2016 [30].

## Supporting information

S1 Database (XLSX)
